# Outcomes of Microsurgical Reconstruction of Post‐Burn Joint Contracture—Systematic Review and Meta‐Analysis

**DOI:** 10.1002/micr.70104

**Published:** 2025-08-15

**Authors:** Abdulaziz Elemosho, Layne N. Raborn Macdonald, Derek E. Bell, Jeffrey E. Janis

**Affiliations:** ^1^ Department of Plastic and Reconstructive Surgery, College of Medicine The Ohio State University Wexner Medical Center Columbus Ohio USA; ^2^ Division of Plastic Surgery University of Rochester Medical Center Rochester New York USA

**Keywords:** burn reconstruction, cicatrix, flap reconstruction, joint contracture, microsurgery, scar management

## Abstract

**Background:**

Contracture recurrence is a common setback to burn reconstruction, especially for severe or large‐area contractures. Flap‐based burn reconstruction has been shown to result in lower recurrent contracture rates. This study aims to summarize and evaluate the outcomes of flap‐based techniques used for post‐burn joint contracture reconstruction.

**Methods:**

A systematic review was performed following PRISMA guidelines. Databases searched included PUBMED, EMBASE, Scopus, and Web of Science. Articles that described the use of flaps with a known blood supply to reconstruct post‐burn contractures of the joints were included. Studies with incomplete data, with multiple anatomic site contracture involvement, case reports, and non‐English articles were excluded. Data on patient demographics, flap type, complications, and contracture resolution were extracted. A proportional meta‐analysis was conducted using the DerSimonian and Laird random‐effects model.

**Results:**

Out of 850 studies screened, 27 met inclusion criteria. Reconstruction of 830 joint contractures was reported. Contractures resolved for 98.9% (*I* ^2^ = 0% [95% CI: 97.7–99.6]) of pedicled and 90.1% (*I* ^2^ = 82.8% [95% CI: 62.7–100]) of free flap reconstructions, recurring in 1.8% (*I* ^2^ = 0% [95% CI: 0.7–3.3]) at sites reconstructed with pedicled flaps and 0.6% (*I* ^2^ = 0% [95% CI: 0.1–1.7]) at sites reconstructed with free flaps. The rates of flap complications were low, with total flap loss reported at 1.5% (*I* ^2^ = 0% [95% CI: 0.6–2.7]) and 2.9% (*I* ^2^ = 37.9% [95% CI: 0.9–5.8]) of the time for pedicled and free flaps, respectively. Partial flap loss was 6.9% (*I* ^2^ = 65.1% [95% CI: 3.4–11.5]) and 5.2% (*I* ^2^ = 65% [95% CI: 1.7–10.4]) for pedicled and free flaps, respectively. No significant difference was identified in rates of contracture resolution (*p* = 0.50), contracture recurrence (*p* = 0.15), total flap loss (*p* = 0.18) or partial flap loss (*p* = 0.31) regardless of the flap type used.

**Conclusions:**

Burn contracture reconstruction using flap‐based techniques shows minimal complications and low rates of contracture recurrence when used for joints. Pedicled and free flap reconstruction of burn contracture sites yield similar outcomes.

## 1 Introduction

Burn contracture is a devastating sequela of burn injury, carrying morbidity for the recovering patient, while also being clinically challenging to correct, requiring an armory of reconstructive skills. Development of post‐burn contracture after a major burn has been reported to be as high as 43%; however, this may vary across treatment facilities depending on the type of burn care and resources available to patient (Kowalske et al. 2003; Schneider et al. 2006). This can be devastating to patients' ability to perform activities of daily living, especially in the setting of joint contractures which inhibit range of motion (Ma et al. 2021). Schneider et al. reported an incidence rate of 38% for shoulder contractures, 34% for elbow contractures, and 22% for knee contractures following acute burn surgery (Schneider et al. 2006). Following initial burn contracture release, recurrence has been estimated to be up to 70%, with the highest recurrences reported for joint contractures (Liu et al. 2024; Balumuka et al. 2015; Carmichael et al. 2005). Identification of successful burn contracture reconstruction techniques for joints is necessary to improve patient morbidity and prevent contracture recurrence.

A successful approach for burn contracture management relies on a thorough understanding of the reconstructive ladder and an appropriate, individualized surgical plan for each patient. Although skin grafts are widely used for burn reconstruction, these are associated with higher contracture rates (Maitani et al. 2021). Local tissue rearrangements may be an effective reconstructive method when healthy nearby tissue is available, but the utility is limited in instances of large or severe burns that necessitate tissue reconstruction using a reliable blood supply. (Verhaegen et al. 2011; Hayashida and Akita 2017) Burn contracture repair using flap‐based reconstruction has been associated with lower rates of re‐contracture comparatively; but it requires a higher technical skill level of the surgeon and can increase morbidity risk for the patient (Verhaegen et al. 2011; Hayashida and Akita 2017).

To date, a summary of the current literature that addresses flap‐based reconstruction of post‐burn joint contractures and assessment of the outcomes is not available. We sought to effectively summarize the current literature on this topic with an anatomically based approach to aid burn reconstruction surgeons in choosing the appropriate reconstructive method. We chose to refine our search further by specifically focusing on joint contractures and ensured that included studies had objective measurements for improvement, including data that reported range of motion outcomes. We hypothesized that flap‐based reconstruction of post‐burn joint contractures would yield low recurrence across joint types, regardless of the flap type used.

## 2 Methods

### 2.1 Search Strategy

We performed a systematic review following the Preferred Reporting Items for Systematic Reviews and Meta‐analysis (PRISMA) guidelines (Page et al. 2021) by searching PUBMED, EMBASE, Scopus, and Web of Science and using several keywords and MeSH headings that include:

(“burns” [MeSH Terms] OR “burns” [All Fields] OR “burn” [All Fields]) AND (“cicatrix” [MeSH Terms] OR “cicatrix” [All Fields] OR “scar” [All Fields]) AND (“contractural” [All Fields] OR “contracture” [MeSH Terms] OR “contracture” [All Fields] OR “contractures” [All Fields] OR “contractured” [All Fields]). The full database‐specific search strategies are available in File S1.

### 2.2 Eligibility Criteria

Articles that were included in this study described the flap‐based repair of a burn contracture that crossed a joint. We defined flap‐based reconstruction as the use of flaps with a blood supply from a named vessel, including free and pedicled flaps. All relevant case series and cohort studies were included. Studies with incomplete data, studies that included contractures that involved two distinct anatomic sites or those that did not involve a joint, studies not written in the English language, case reports, and studies involving contracture repair with local tissue rearrangements without an axial blood supply were excluded.

### 2.3 Screening, Selection, and Data Extraction

Screening was independently conducted using Covidence systematic review software by two independent authors (A.E and L.R.M.), and conflicts were resolved by consensus and adjudication by the senior author (J.E.J.), if needed. Results from the initial search were compiled, with duplicate studies removed. After title and abstract screening, full‐length articles were reviewed with reasons provided for each exclusion. The selected articles were reviewed, and data describing the authors of the study, year of publication, type of study, total number of patients, demographics of patients, follow‐up duration, type of reconstruction, time from initial reconstruction, reconstructive details, and reconstructive outcomes including flap loss and contracture recurrence were collected.

### 2.4 Risk of Bias and Study Quality Assessment

The methodological quality of all included studies was independently evaluated by two authors. Given that no randomized controlled trials were identified, the Methodological Index for Non‐Randomized Studies (MINORS) tool was applied to assess study quality (Slim et al. 2003). To explore the presence of potential publication bias, funnel plots were generated based on effect sizes of reported outcomes.

### 2.5 Data Analysis and Synthesis

Descriptive statistics were used to summarize continuous outcomes. All included studies utilized individual patients as the unit of analysis. Means were pooled from across studies, and information about the spread was provided using “range of means.”

Statistical analyses and graph generation were conducted using MedCalc statistical software version 23.0.5 (MedCalc Software bvba, Ostend, Belgium). Data were pooled using a random‐effects model with the DerSimonian and Laird estimator (DerSimonian and Laird 2015). Incidences and corresponding 95% confidence intervals were calculated using the exact binomial method, with proportions stabilized via the Freeman–Tukey double arcsine transformation to describe key complication rates, including total and partial flap loss, venous congestion, and tip necrosis when available. Heterogeneity for each outcome was assessed using the I (Schneider et al. 2006) statistic, with values of 0%–25% indicating low heterogeneity, 26%–50% moderate, and over 50% high (Higgins et al. 2003). Chi‐squared tests were applied to analyze weighted proportions, with statistical significance defined as *p* < 0.05. Sensitivity analyses were not performed, as they are not typically applicable in proportional meta‐analyses of single‐arm outcomes. The pooled estimates reflect the overall incidence of events across studies without comparative arms, and heterogeneity was addressed using a random‐effects model.

### 2.6 Outcome Measures

Improvement of contracture was defined as improved joint range of motion following reconstruction, as reported in each individual study. Outcome measures were categorized by anatomic site as follows: for hand contractures, clinical improvement in activities of daily living was used (Davami and Pourkhameneh 2011); for axillary contractures, either clearly stated clinical improvement or improvement in the preoperative range of abduction (Chen et al. 2017); for neck contractures, includes clearly stated clinical improvement, cervicomental angle of less than 110° or postoperative neck extension exceeding the horizontal plane (> 100°) (Angrigiani 1994; Wang et al. 2012); and for elbow and knee contractures, either clearly stated clinical improvement or improvements in the preoperative arc of flexion–extension (Chang et al. 2021; Karagoz et al. 2011).

## 3 Results

### 3.1 Study Characteristics and Demographics

**FIGURE 1:**
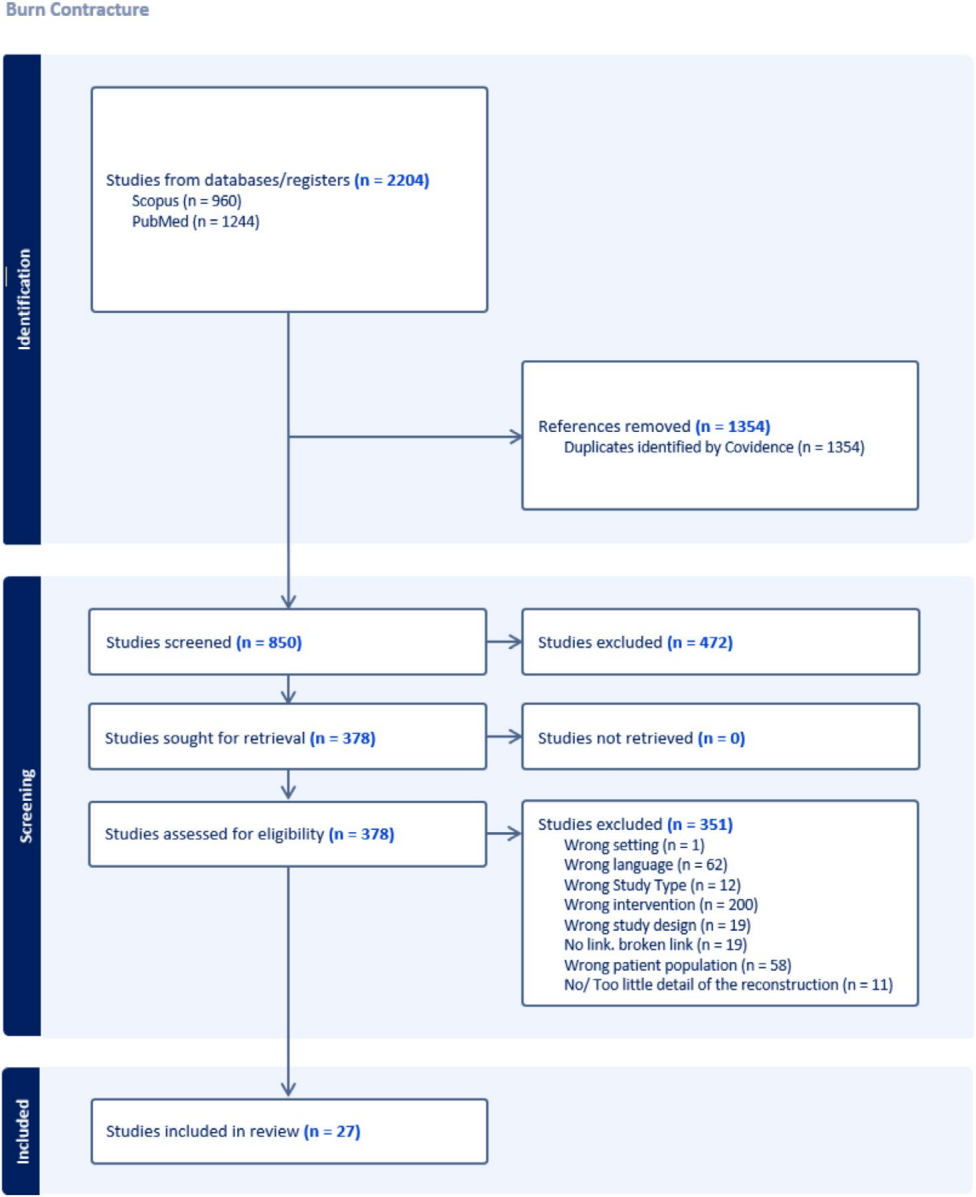
PRISMA flow chart.

Out of the 850 studies screened, 27 studies met final inclusion criteria (Figure 1) (Davami and Pourkhameneh 2011; Chen et al. 2017; Angrigiani 1994; Wang et al. 2012; Chang et al. 2021; Karagoz et al. 2011; Yildirim et al. 2003; El‐Khatib et al. 2002; Er and Uçar 2005; Eski et al. 2007; Feng et al. 2010; Gousheh et al. 2008; Hafezi et al. 2008; Hassanpour et al. 2007; Nişanci et al. 2002; Sever et al. 2012; Uygur et al. 2009; Vinh et al. 2007, 2009; Loghmani et al. 2013; Angrigiani et al. 2017; Bali et al. 2021; Moroz et al. 2001; Mun et al. 2007; Sarkar et al. 2014; Woo and Seul 2001; Grishkevich 2012). There were 25 retrospective studies and 2 case series that were published between 1998 and 2021. Table 1 summarizes the characteristics of included studies, and a full list of references for these articles can be found in File S2.

**TABLE 1:** List of included articles.

Authors	Year	Number of contracture repaired	Location of contracture	Flap used	MINORS score
Angrigiani (1994)	1994	86	Neck	Scapular and parascapular free flap	10
Woo and Seul (2001)	2001	18	Hand		10
Moroz et al. (2001)	2001	63	Neck	Deltoid, radial, scapular free flaps	12
El‐Khatib et al. (2002)	2002	13	Elbow	Pedicled adipofascial flap	10
Nişanci et al. (2002)	2002	32	Axilla	Scapular and parascapuar flaps	12
Yildirim et al. (2003)	2003	8	Knee	Free ALT musculocutaneous flap	10
Er and Uçar (2005)	2005	15	Axilla	Thoracodorsal perforator‐based cutaneous island flap	10
Eski et al. (2007)	2007	14	Hand	First dorsal metacarpal artery flap	10
Hassanpour et al. (2007)	2007	12	Elbow	Bipedicled flap from scar tissue	10
Mun et al. (2007)	2007	12	Neck	Thin thoracodorsal artery perforator flap	12
Vinh et al. (2007)	2007	30	Neck	Pedicled supraclavicular artery‐based flap	10
Gousheh et al. (2008)	2008	42	Hand	Super‐thin abdominal skin pedicle flap	12
Hafezi et al. (2008)	2008	15	Neck	Extended vertical trapezius fasciocutaneous flap	12
Uygur et al. (2009)	2009	9	Elbow	Thoracodorsal artery perforator flap	11
Vinh et al. (2009)	2009	101	Neck	Pedicled supraclavicular artery‐based flap	10
Feng et al. (2010)	2010	8	Hand, axilla, neck	Medial thigh perforator flap	10
Davami and Pourkhameneh (2011)	2011	53	Hand	Axial groin flap	11
Karagoz et al. (2011)	2011	6	Elbow	Lateral intercostal artery perforator‐based pedicled reverse thoraco‐abdominal flap	10
Grishkevich (2012)	2012	32	Neck	Cervicothoracic flap based on superficial cervical artery perforator	12
Sever et al. (2012)	2012	7	Elbow	Pedicled thoracodorsal artery perforator flap	8
Wang et al. (2012)	2012	68	Neck	Pedicled deltopectoral flaps	10
Loghmani et al. (2013)	2013	32	Neck	Pedicled supraclavicular artery‐based flap	12
Sarkar et al. (2014)	2014	11	Neck	ALT	12
Angrigiani et al. (2017)	2017	99	Neck	Extended circumflex scapular artery free flap	12
Chen et al. (2017)	2017	10	Axilla	Free ALT musculocutaneous flap	10
Chang et al. (2021)	2021	22	Elbow, knee	Free thoracodorsal artery flap	14
Bali et al. (2021)	2021	13	Neck, knee	ALT flap	12

Abbreviation: ALT flap, anterolateral thigh flap.

There were 830 contractures identified which underwent flap‐based reconstruction. Anatomic areas included the hand (including dorsal and volar surfaces), cubital fossa, axilla, neck (anterior and lateral) and popliteal fossa. Other anatomic areas including groin and wrist were sparsely reported in the literature; hence, not included in this study.

Mean age of included patients was 27.3 years (IQR: 11.3–51 years) and mean follow‐up duration was 23.8 months (IQR: 6.6–108 months). Males comprised 63.7% of the study population. Overall average time between initial reconstruction and contracture development/repair was 38.3 months (IQR: 8.3–107 months).

### 3.2 Risk of Bias Assessment

The 27 included studies were assessed using the MINORS criteria. Scores ranged from 8 to 14, with a median score of 11. The most common methodological limitations were lack of prospective data collection, small study size, and unclear reporting of follow‐up duration. In contrast, all studies demonstrated clearly stated aims, appropriate endpoints, and unbiased assessment of outcomes. Individual MINORS scores for each study are provided in Table 1.

### 3.3 Hand

A total of 128 contractures of the hand and fingers were released and repaired. The axial groin flap was the most used flap at 40.6% (*n* = 52) followed by the super‐thin abdominal skin pedicle flap (based on the superficial inferior epigastric artery) at 32.0% (*n* = 41). The first dorsal metacarpal artery flap [10.9%, *n* = 14], dorsalis pedis free flap [5.5%, *n* = 7] and medial thigh perforator flap [1.6%, *n* = 2] were the least frequently utilized. Other flaps used included the posterior interosseous free flap at 3.1% (*n* = 4), free arterialized venous flap at 3.1% (*n* = 4), free radial forearm flap at 1.6% (*n* = 2) and “fill‐up webspace” flap (a modified dorsalis pedis artery flap) at 1.6% (*n* = 2). All flaps survived, and contracture improvement was exhibited in 99.3% (*n* = 127) of cases. There were no instances of flap venous congestion or reports of significant donor‐site morbidity. Partial flap loss was observed in 5.4% (*n* = 7) of contractures repaired. The contracture recurrence rate was 0.7% (*n* = 1), but no contractures required secondary revision procedures (Table 2).

**TABLE 2:** Summary of flap complications by location repaired.

Location (number of flaps)	Flap types used	Total flap loss	Partial flap loss	Flap venous congestion	Donor site morbidity
Hand (128)	Axial groin flap, super‐thin abdominal skin pedicle flap, first dorsal metacarpal artery flap, dorsalis pedis free flap, posterior interosseous free flap, arterialized venous flap, free radial forearm flap, “fill‐up webspace” flap	0	5.4%	0	0
Elbow (52)	Thoracodorsal artery perforator flap, pedicled adipofascial flap, bipedicled flap, lateral intercostal artery perforator‐based reverse thoraco‐abdominal flap	0	5.8%	9.6%	1.9%
Axilla (62)	Scapular island flap, parascapular flap, thoracodorsal perforator cutaneous island flap, anterolateral thigh flap, medial thigh perforator flap	0	1.6%	0	0
Neck (558)	Supraclavicular flap, deltopectoral flap, superficial cervical artery perforator flap, trapezius fasciocutaneous flap, free ALT flap, Free thoracodorsal artery perforator flap	2.5%	4.8%	4.6%	1.9%
Knee (30)	Thoracodorsal perforator artery flap, anterolateral thigh flap	0	20%	0	0

### 3.4 Axilla

A total of 62 axillary contractures were repaired. Scapular flaps were used in 43.5% (*n* = 27) of contractures, including 30.6% (*n* = 19) scapular island flaps and 12.9% (*n* = 8) parascapular flaps. Thoracodorsal perforator‐based cutaneous island flaps were used in 29.0% (*n* = 18) of reported contractures; anterolateral thigh (ALT) flaps in 24.2% (*n* = 15); and medial thigh perforator flaps in 3.3% (*n* = 2).

All flaps survived, and contracture improvement was observed in all (*n* = 62) cases. No instances of flap venous congestion or significant donor‐site morbidity were reported. Partial flap loss occurred in 1.6% (*n* = 1) of flaps. No contracture recurrence or need for secondary revision procedures was reported (Table 2).

### 3.5 Neck

A total of 558 neck contractures were released and repaired. Supraclavicular flaps (island and skin pedicled) were used in 74.5% (*n* = 416) of contractures, deltopectoral flaps in 12.4% (*n* = 69), superficial cervical artery perforator flaps in 5.7% (*n* = 32), trapezius fasciocutaneous flaps in 2.3% (*n* = 13), free ALT flaps in 2.9% (*n* = 16), and free thoracodorsal artery perforator flaps in 2.2% (*n* = 12) of reported reconstructions.

Flap survival was observed in 97.5% (*n* = 544) of cases. There was a reported total flap loss of 2.5% (*n* = 14), venous congestion in 4.1% (*n* = 23), and partial flap loss in 4.8% (*n* = 27). Contracture resolution was achieved in 96.2% (*n* = 537) of patients, with no reported recurrence. Elective secondary procedures were reported in two studies and included flap debulking and scar revisions including *z*‐plasty (Wang et al. 2012; Sunil et al. 2015). (Table 2).

### 3.6 Elbow

A total of 52 elbow contractures were repaired. The mean repair timing was 26.9 months (IQR: 8.3–43 months) following the initial burn injury. The thoracodorsal artery perforator flap was used for 40.4% (*n* = 21) of elbow contracture repairs, followed by the pedicled adipofascial flap (based on the ulnar artery) for 25% (*n* = 13), the bipedicled flap (based on the posterior radial collateral artery [PRCA]) for 23.1% (*n* = 12) and the lateral intercostal artery perforator‐based pedicled reverse thoraco‐abdominal flap for 11.5% (*n* = 6) of reported contracture reconstructions. All (*n* = 52) flaps survived, and contractures were successfully addressed. Venous congestion occurred in 9.6% (*n* = 5) of cases, with a partial flap loss rate of 5.8% (*n* = 3) and donor‐site morbidity, specifically the development of a seroma, reported in 1.9% (*n* = 1). Three (5.8%) of the contractures repaired needed secondary debulking to improve aesthetics; however, there was no reported contracture recurrence (Table 2).

### 3.7 Knee

A total of 30 contractures of the popliteal fossa were repaired. The thoracodorsal perforator artery flap was used in 56.7% (*n* = 17) of cases, while the ALT flap was used in 43.3% (*n* = 13) of cases. All flaps survived, and there were no reported instances of flap venous congestion or donor‐site morbidity. Partial flap loss occurred in 20.0% (*n* = 6) of contractures. Contracture resolution was observed in 63.3% (*n* = 19) of cases, with no reported recurrences. Additional procedures, including debulking and scar revisions, were performed in 16.7% (*n* = 5) of contractures (Table 2).

### 3.8 Overall Complications

The rate of pedicled flap loss was 1.5% (*I* ^2^ = 0% [95% CI: 0.6–2.7]) and the rate of free flap loss was 2.9% (*I* ^2^ = 37.9% [95% CI: 0.9–5.8]). Partial flap loss was 6.9% (*I* ^2^ = 65.1% [95% CI: 3.4–11.5]) for pedicled and 5.2% (*I* ^2^ = 65.0% [95% CI: 1.7–10.4]) for free flaps. Flap venous congestion rate was only reported for pedicled flaps and was 7.5% (*I* ^2^ = 81.4%) Table 3.

**TABLE 3:** Summary of overall complications.

Flap type	Total flap loss	Partial flap loss	Flap venous congestion
Free flaps (353)	2.9% [95% CI: 0.9–5.8]	5.2% [95% CI: 1.7–10.4]	—
Pedicled flaps (477)	1.5% [95% CI: 0.6–2.7]	6.9% [95% CI: 3.4–11.5]	7.5% [95% CI: 1.3–17.9]

Contracture resolution rate was found to be 98.9% (*I* ^2^ = 0%) for pedicled and 92.2% (*I* ^2^ = 82.8%) for free flaps. Details of range of motion assessment are presented in Table 4. Contracture recurrence rate was 1.8% (*I* ^2^ = 0% [95% CI: 0.7–3.3]) for pedicled flaps and 0.6% (*I* ^2^ = 0% [95% CI: 0.1–1.7]) for free flaps.

**TABLE 4:** Reported range of movement.

Article	Anatomic site	ROM assessment	Reported improvement in the range of motion	Reported rate of flap debulking or scar revision
Angrigiani (1994)	Neck	Cervicomental angle < 110° satisfactory	83 of 86 contractures (96%)	33% (*n* = 28) (debulking)
Angrigiani et al. (2017)	Neck	Cervicomental angle < 110° satisfactory	90 of 99 contractures (90.9%)	60% (*n* = 45) (debulking)
Wang et al. (2012)	Neck	Neck extension exceeding the horizontal plane (> 100°)	59 of 68 contractures (86.8%)	25% (*n* = 17) scar revisions
Loghmani et al. (2013)	Neck	Subjective reporting	29 of 32 contractures (90.6%)	75% (*n* = 24)
Sarkar et al. (2014)	Neck	Improvement in preoperative cervicomandibular angle, neck extension, neck rotation, and lateral bending	Average cervicomandibular angle was improved by 59.28° (preop, 55.72°; postop, 115°). Average increase in extension of 35.09° (preop, 18.09°; postop, 53.18°) Average increase in rotation of 46.09° (preop, 29.63°; postop, 75.72°) Average increase in lateral flexion of 23.95° (preop, 17.5°; postop, 41.45°)	Not reported
Grishkevich (2012)	Neck	Subjective	25 of 32 contractures (78.1%) (good outcome in 1‐stage reconstruction) 7 of 32 contractures (21.9%) (good outcome after multi‐stage reconstruction)	Not reported
Moroz et al. (2001)	Neck	Subjective	Not reported	31.7% (*n* = 20)
Mun et al. (2007)	Neck	Cervicomental angle (stated normal range 106°–120°) Neck extension	Average cervicomental angle increased by 48° (preop, 149°; postop, 101°)—satisfactory in 12 of 12 patients (100%) Average increase in extension of 19° (preop, 38°; postop, 57°)	Not reported
Vinh et al. (2007)	Neck	Subjective	Not reported	Not reported
Vinh et al. (2009)	Neck	Subjective	Not reported	Not reported
Hafezi et al. (2008)	Neck	Subjective	Not reported	Not reported
Bali et al. (2021)	Knee, neck	Subjective	Not reported	Not reported
Feng et al. (2010)	Hand, axilla, neck	Neck extension and flexion, wrist extension and flexion, shoulder extension, flexion, and abduction	Neck (mean extension: preop of 21.5° to 67.5° postop; mean flexion: preop of 17.5° to 66° postop) Wrist (mean extension: preop 27° to 77.5° postop, mean flexion: preop 25° to 77.5° postop) Shoulder (mean extension: preop 24.2° to 44.5° postop; mean flexion: preop 88.7° to 155° postop; mean abduction range: preop 73.8° to 136.3°)	Not reported
El‐Khatib et al. (2002)	Elbow	Subjective (functional deficit and aesthetic outcomes)	13 of 13 contractures (100%)	Not reported
Hassanpour et al. (2007)	Elbow	Preoperative versus postoperative extension lag	Average extension lag preop 66.5° to postop 5.4°	Not reported
Uygur et al. (2009)	Elbow	Graded as *excellent* (full range of motion), *good* (significant improvement in range of motion), *average* (some improvement in range of motion), or *below average* (no improvement in range of motion)	Excellent: 5 contractures Good: 4 contractures	Not reported
Karagoz et al. (2011)	Elbow	Preoperative versus postoperative extension lag	Average extension lag preop 68.3° to postop 3.3°	Not reported
Sever et al. (2012)	Elbow, axilla	Varied reporting: elbow and arm extension, arm abduction	Not available	Not available
Chang et al. (2021)	Elbow, knee	Preoperative to postoperative flexion–extension range	Mean increase ROM was 18.6° (range 0°–70°). ROM increased in 14 out of 25 contractures (56%)	Not reported
Chen et al. (2017)	Axilla	Preoperative to postoperative abduction range	Average increase in ROM of shoulder abduction was 86° for all contractures (preop 62°, postop 148°)	Not reported
Nişanci et al. (2002)	Axilla	Objective measurement not reported	Objective measurement not reported	Not reported
Er and Uçar (2005)	Axilla	Preoperative to postoperative abduction range	Average preop 46.6° to postop 159°	Not reported
Yildirim et al. (2003)	Knee	Preoperative to postoperative flexion–extension range	Reported by cases (varied)	Not reported
Eski et al. (2007)	Hand	Clinical and functional improvement	14 of 14 contractures (100%).	Not reported
Davami and Pourkhameneh (2011)	Hand	Improvement in the activity of daily living (ADL)	40 out of 53 contractures (75.7%) had a satisfactory improvement in ADL following initial reconstruction	Not reported
Gousheh et al. (2008)	Hand	Subjective assessment	42 out of 42 contractures (100%)	Not reported
Woo and Seul (2001)	Hand	Jebsen test Grip strength Pinch test	Varied reporting	Not reported

When pedicled and free flap outcomes were directly compared, there was no difference in the rate of total flap loss (1.5% vs. 2.9% *p* = 0.18), partial flap loss (6.9% vs. 5.2% *p* = 0.31), contracture resolution (98.9% vs. 92.2% *p* = 0.50) or contracture recurrence rates (1.8% vs. 0.6% *p* = 0.15) respectively.

## 4 Discussion

Successful burn contracture reconstruction necessitates consideration of available healthy tissue, reconstructive morbidity, and mitigation of the risk of recurrence. Studies have reported that flap reconstruction is the best method to correct contractures when aiming to prevent recurrence (Verhaegen et al. 2011; Hayashida and Akita 2017). This is particularly important when contractures involve joints, areas that a contracture can diminish or prevent functional capabilities. In this study, 830 burn contractures were identified which were reconstructed with 477 pedicled (57.5%) and 353 free flaps (42.5%). Overall, an improvement in joint movement was seen in more than 90% of the sites reconstructed.

We demonstrated that the rates of re‐contracture were 1.8% for repairs with pedicled flaps and 0.6% for repairs with free flaps, with a mean follow‐up of 23.8 months. These findings were similar to rates reported by Lui et al., who reported a re‐contracture rate of 0.6% following free flap reconstruction of scars (Liu et al. 2024). Further, hand contractures recurred in 0.7% (*n* = 1) of contractures from our included studies, which indicated better results than those previously reported following split‐thickness skin graft reconstruction (17%) (Sunil et al. 2015). Currently, literature reports that postoperative recurrent contracture can develop as far out as 17–24 months (Balumuka et al. 2015; Carmichael et al. 2005). With this consideration, our recurrence rate was less than that reported following graft reconstruction, providing further support for the utility of flap‐based reconstruction for post‐burn joint contractures. This study uniquely reports the rate of joint re‐contractures following pedicled and free flap reconstruction of burn contractures, successfully demonstrating excellent outcomes compared to non‐flap based reconstructive techniques.

Flap reconstruction carries a risk of additional procedures, particularly if flap loss occurs or if the flap is aesthetically disfiguring. In terms of flap failure rates, 1.5% of pedicled flaps and 2.9% of free flaps were reported to be non‐viable in this study, the instance of this being similar between pedicled and free flaps. Liu et al. previously reported a total flap loss rate of 3.8% for contracture repairs using free flaps, which is similar to our findings (Liu et al. 2024). The rate of partial flap loss in free flap reconstructions was comparable to what has been reported (Liu et al. 2024). It is important to note that the rate of free flap loss is indirectly related to surgeon skill (Bleasdale et al. 2015), indicating that microsurgically trained surgeons are an important resource for burn centers that seek to provide optimal joint contracture reconstruction.

Our study found that most elective secondary procedures were performed for the elbows, neck, and knees. Debulking procedures were utilized to improve contour, particularly in instances when the chosen reconstructive flap was thicker than the surrounding tissue. Additionally, procedures to improve scar hypertrophy and keloids were also reported. Silicone has been a commonly reported substance that could be utilized to decrease the need for secondary procedures in the early stages of wound healing if a free flap is planned (Balumuka et al. 2015). Overall, aesthetically targeted revision procedures are more common for exposed reconstructed areas, and patients should be aware of this before choosing this reconstructive method (Pradier et al. 2007; Petro and Niazi 2010). Unlike flap loss, aesthetic refinements can be addressed non‐urgently and be scheduled around functional improvement.

There are numerous emerging techniques aimed at reducing the risk of contracture rates aside from flaps or autografts. These include the use of acellular dermal matrices, such as Integra, Dermacell, and Kerecis, as well as various allografts (Dilek et al. 2024). These techniques involve staging grafts with an intermediary substance, which has been shown to significantly diminish the risk of re‐contracture. Although these methods are promising, they are still in the early stages of study and their long‐term impact remains uncertain, with limited data available on their effects on burn contracture prevention (Pradier et al. 2007). These advancements may shift clinical practice toward reducing the morbidity associated with flap reconstruction. However, further data is needed to fully understand how these innovations compare to flaps and grafts.

Our study has limitations owed largely to issues with inconsistent reporting of source data used to perform this systematic review and meta‐analysis and lack of uniform anatomic descriptions. For example, some studies did not make a clear distinction between anterior neck and lateral neck contracture repairs. This did not allow us to separate the outcomes and discretely analyze. Additionally, some studies did not differentiate secondary flap procedures performed, as some patients may have had these procedures to improve cosmesis, for example, debulking or to improve mobility, for example, additional *z*‐plasty. Due to the complexities of burn reconstructions, variation in surgical techniques may exist, potentially confounding any reported negative outcomes. Another limitation of this study is the lack of standardized reporting for joint range of motion outcomes. Most studies noted that range of motion improved following reconstruction; however, this was assessed in different ways according to each study. Further, comparisons across joint sites could not be made due to the different study designs of the included studies. Although justified by severe limitation of function leading to reconstruction of contracture with flaps (Chang et al. 2021), the time from initial reconstruction to this definitive reconstruction may be short based on senior authors experience. This may have potentially overestimated the rate of contracture development using alternative techniques. Finally, some studies may have underreported their negative outcomes, as we could not compare outcomes of free versus pedicled flaps on venous congestion and contracture recurrence due to incomplete reporting. Additionally, the only anatomic site that total flap loss was reported for was the neck, which could indicate reporting bias in the literature. From the authors own experience, there were some flap reconstruction options that were not cited in the literature, for example, the use of a pedicled latissimus dorsi flap for axillary burn contracture reconstruction. This points to another limitation of this analysis, which is that it may not include all methods of reconstruction available to the burn surgeon.

## 5 Conclusions

Through this study, supporting evidence is provided for the use of flap‐based reconstruction in the setting of post‐burn contracture management. Although the morbidity of a flap may be more than grafting or local tissue rearrangement, the rates of re‐contracture and risk are low enough to support their use, especially for joint reconstruction, which requires adequate range of motion for function.

## Author Contributions

**Abdulaziz Elemosho:** conception and design, acquisition and screening of data, analysis and interpretation of data, drafting the article or revising it critically for important intellectual content, review and editing. **Layne N. Raborn Macdonald:** conception and design, acquisition and screening of data, analysis and interpretation of data, drafting the article or revising it critically for important intellectual content, review and editing. **Derek E. Bell:** conception and design, acquisition of data, analysis and interpretation of data, drafting the article or revising it critically for important intellectual content, review and editing, final approval of the version to be published. **Jeffrey E. Janis:** conception and design, acquisition of data, analysis and interpretation of data, drafting the article or revising it critically for important intellectual content, review and editing, final approval of the version to be published. All authors have read and approved the final manuscript.

## Conflicts of Interest

Dr. Janis receives royalties from Thieme and Springer Publishing. Dr. Bell is a key opinion leader, funded researcher and speaker for AVITA Medical Inc. and a consultant for PolyNovo's NovoSorb BTM product. He also has been awarded a research grant from Spectral MD. All other authors declare no conflicts of interest.

## Supporting information


**File S1:** micr70104‐sup‐0001‐SDC1.docx.

**File S2:** micr70104‐sup‐0002‐SDC2.docx.

## Data Availability

The data that support the findings of this study are publicly available and in the Supporting Information as well.

## References

[micr70104-bib-0001] Angrigiani, C. 1994. “Aesthetic Microsurgical Reconstruction of Anterior Neck Burn Deformities.” Plastic and Reconstructive Surgery 93, no. 3: 507–518. 8115505

[micr70104-bib-0002] Angrigiani, C. , G. Artero , C. Sereday , R. K. Khouri Jr. , and Z. P. French . 2017. “Refining the Extended Circumflex Scapular Flap for Neck Burn Reconstruction: A 30‐Year Experience.” Journal of Plastic, Reconstructive & Aesthetic Surgery 70, no. 9: 1252–1260. 10.1016/j.bjps.2017.05.047 28662866

[micr70104-bib-0003] Bali, Z. U. , B. Özkan , Y. Keçeci , et al. 2021. “Reconstruction of Burn Contractures With Free Anterolateral Thigh Flap in Various Anatomic Sites.” Ulusal Travma ve Acil Cerrahi Dergisi 27, no. 4: 337–343. 33884605 10.14744/tjtes.2020.89195

[micr70104-bib-0004] Balumuka, D. D. , G. W. Galiwango , and R. Alenyo . 2015. “Recurrence of Post Burn Contractures of the Elbow and Shoulder Joints: Experience From a Ugandan Hospital.” BioMed Central Surgery 15: 103. 10.1186/s12893-015-0089-y. 26353814 PMC4564967

[micr70104-bib-0005] Bleasdale, B. , S. Finnegan , K. Murray , S. Kelly , and S. L. Percival . 2015. “The Use of Silicone Adhesives for Scar Reduction.” Advanced Wound Care 4, no. 7: 422–430. 10.1089/wound.2015.0625. PMC4486716 26155385

[micr70104-bib-0006] Carmichael, K. D. , S. C. Maxwell , and J. H. Calhoun . 2005. “Recurrence Rates of Burn Contracture Ankle Equinus and Other Foot Deformities in Children Treated With Ilizarov Fixation.” Journal of Pediatric Orthopedics 25, no. 4: 523–528. 10.1097/01.bpo.0000161093.31092.c4. 15958908

[micr70104-bib-0007] Chang, L. S. , Y. H. Kim , and S. W. Kim . 2021. “Reconstruction of Burn Scar Contracture Deformity of the Extremities Using Thin Thoracodorsal Artery Perforator Free Flaps.” ANZ Journal of Surgery 91, no. 9: E578–E583. 10.1111/ans.16640. 33792136

[micr70104-bib-0008] Chen, H. C. , K. P. Wu , C. I. Yen , et al. 2017. “Anterolateral Thigh Flap for Reconstruction in Postburn Axillary Contractures.” Annals of Plastic Surgery 79, no. 2: 139–144. 10.1097/SAP.0000000000001097. 28570453

[micr70104-bib-0009] Davami, B. , and G. Pourkhameneh . 2011. “Correction of Severe Postburn Claw Hand.” Techniques in Hand & Upper Extremity Surgery 15, no. 4: 260–264. 10.1097/BTH.0b013e3182245b56. 22105641

[micr70104-bib-0010] DerSimonian, R. , and N. Laird . 2015. “Meta‐Analysis in Clinical Trials Revisited.” Contemporary Clinical Trials 45, no. Pt A: 139–145. 10.1016/j.cct.2015.09.002. 26343745 PMC4639420

[micr70104-bib-0011] Dilek, Ö. F. , K. Z. Sevim , and O. N. Dilek . 2024. “Acellular Dermal Matrices in Reconstructive Surgery; History, Current Implications and Future Perspectives for Surgeons.” World Journal of Clinical Cases 12, no. 35: 6791–6807. 10.12998/wjcc.v12.i35.6791. 39687641 PMC11525903

[micr70104-bib-0012] El‐Khatib, H. A. , T. A. Mahboub , and T. A. Ali . 2002. “Use of an Adipofascial Flap Based on the Proximal Perforators of the Ulnar Artery to Correct Contracture of Elbow Burn Scars: An Anatomic and Clinical Approach.” Plastic and Reconstructive Surgery 109, no. 1: 130–136. 10.1097/00006534-200201000-00022. 11786804

[micr70104-bib-0013] Er, E. , and C. Uçar . 2005. “Reconstruction of Axillary Contractures With Thoracodorsal Perforator Island Flap.” Burns 31, no. 6: 726–730. 10.1016/j.burns.2005.02.014. 16129226

[micr70104-bib-0014] Eski, M. , M. Nisanci , and M. Sengezer . 2007. “Correction of Thumb Deformities After Burn: Versatility of First Dorsal Metacarpal Artery Flap.” Burns 33, no. 1: 65–71. 10.1016/j.burns.2006.04.030. 17095165

[micr70104-bib-0015] Feng, C. H. , J. Y. Yang , S. S. Chuang , C. Y. Huang , Y. C. Hsiao , and C. Y. Lai . 2010. “Free Medial Thigh Perforator Flap for Reconstruction of the Dynamic and Static Complex Burn Scar Contracture.” Burns 36, no. 4: 565–571. 10.1016/j.burns.2009.07.005. 19819077

[micr70104-bib-0016] Gousheh, J. , E. Arasteh , and P. Mafi . 2008. “Super‐Thin Abdominal Skin Pedicle Flap for the Reconstruction of Hypertrophic and Contracted Dorsal Hand Burn Scars.” Burns 34, no. 3: 400–405. 10.1016/j.burns.2007.03.025. 17822855

[micr70104-bib-0017] Grishkevich, V. M. 2012. “Unilateral Cervical Burn Scar Deformity Elimination With Contralateral Cervicothoracic Flap – A New Approach.” Journal of Burn Care & Research: Official Publication of the American Burn Association 33, no. 2: e26–e31. 10.1097/BCR.0b013e3182331d4c. 22079901

[micr70104-bib-0018] Hafezi, F. , B. Naghibzadeh , M. Pegahmehr , N. Boddouhi , and A. Nouhi . 2008. “Extended Vertical Trapezius Fasciocutaneous Flap (Back Flap) in Face and Neck Burn Scar Reconstruction.” Annals of Plastic Surgery 61, no. 4: 441–446. 10.1097/SAP.0b013e31815f128a. 18812718

[micr70104-bib-0019] Hassanpour, S. E. , S. Motamed , and M. Ghazisaidi . 2007. “Treatment of Wide Scar Contracture of Antecubital Fossa With Bipedicle Flap From Scar Tissue.” Burns 33, no. 2: 236–240. 10.1016/j.burns.2006.06.023. 17141418

[micr70104-bib-0020] Hayashida, K. , and S. Akita . 2017. “Surgical Treatment Algorithms for Post‐Burn Contractures.” Burns and Trauma 5: 9. 10.1186/s41038-017-0074-z. 28317000 PMC5348756

[micr70104-bib-0021] Higgins, J. P. , S. G. Thompson , J. J. Deeks , and D. G. Altman . 2003. “Measuring Inconsistency in Meta‐Analyses.” British Medical Journal (Clinical Research Edition) 327, no. 7414: 557–560. 10.1136/bmj.327.7414.557. PMC192859 12958120

[micr70104-bib-0022] Karagoz, H. , F. Eren , and E. Ulkur . 2011. “Use of the Lateral Intercostal Artery Perforator‐Based Pedicled Reverse Thoraco‐Abdominal Flap for Treatment of Antecubital Burn Contractures.” Burns 37, no. 1: 134–138. 10.1016/j.burns.2010.03.010. 20537802

[micr70104-bib-0023] Kowalske, K. , R. Holavanahalli , M. Serghio , et al. 2003. “Contractures Following Burn Injury in Children and Adults—A Multicenter Report.” Journal of Burn Care and Rehabilitation 24, no. Suppl 2: S85.

[micr70104-bib-0024] Liu, H. Y. , M. Alessandri‐Bonetti , J. A. Kasmirski , G. M. Stofman , and F. M. Egro . 2024. “Free Flap Failure and Contracture Recurrence in Delayed Burn Reconstruction: A Systematic Review and Meta‐Analysis.” Plastic and Reconstructive Surgery. Global Open 12, no. 8: e6026. 10.1097/GOX.0000000000006026. 39129842 PMC11315556

[micr70104-bib-0025] Loghmani, S. , M. Eidy , M. Mohammadzadeh , A. Loghmani , and F. Raigan . 2013. “The Supraclavicular Flap for Reconstruction of Post‐Burn Mentosternal Contractures.” Iranian Red Crescent Medical Journal 15, no. 4: 292–297. 10.5812/ircmj.1600. 24083000 PMC3785901

[micr70104-bib-0026] Ma, Z. , R. Mo , C. Chen , X. Meng , and Q. Tan . 2021. “Surgical Treatment of Joint Burn Scar Contracture: A 10‐Year Single‐Center Experience With Long‐Term Outcome Evaluation.” Annals of Translational Medicine 9, no. 4: 303. 10.21037/atm-20-4947. 33708930 PMC7944269

[micr70104-bib-0027] Maitani, K. , K. Tomita , M. Taminato , and T. Kubo . 2021. “Effectiveness of Skin Graft in the Chest for Postburn Cervical Contractures.” Plastic and Reconstructive Surgery. Global Open 9, no. 11: e3929. 10.1097/GOX.0000000000003929. 35028260 PMC8751771

[micr70104-bib-0028] Moroz, V. , A. Yudenich , T. Kafarov , P. Sarygin , and V. Sharobaro . 2001. “Reconstruction of Extensive Postburn Scar Deformities and Contractures of the Neck Using Expanded and Nonexpanded Free Tissue Transfer.” European Journal of Plastic Surgery 24: 217–220.

[micr70104-bib-0029] Mun, G. H. , B. J. Jeon , S. Y. Lim , et al. 2007. “Reconstruction of Postburn Neck Contractures Using Free Thin Thoracodorsal Artery Perforator Flaps With Cervicoplasty.” Plastic and Reconstructive Surgery 120, no. 5: 1524–1532. 18040183 10.1097/01.prs.0000282040.39007.1d

[micr70104-bib-0030] Nişanci, M. , E. Er , S. Işik , and M. Sengezer . 2002. “Treatment Modalities for Post‐Burn Axillary Contractures and the Versatility of the Scapular Flap.” Burns 28, no. 2: 177–180. 10.1016/S0305-4179(01)00090-0. 11900943

[micr70104-bib-0031] Page, M. J. , J. E. McKenzie , P. M. Bossuyt , et al. 2021. “The PRISMA 2020 Statement: An Updated Guideline for Reporting Systematic Reviews.” British Medical Journal (Clinical Research Edition) 372: n71. 10.1136/bmj.n71. PMC8005924 33782057

[micr70104-bib-0032] Petro, J. A. , and Z. Niazi . 2010. “Burn Reconstruction.” In Plastic Surgery Secrets Plus, edited by J. Weinzweig , 2nd ed., 665–673. Mosby. 10.1016/B978-0-323-03470-8.00103-4.

[micr70104-bib-0033] Pradier, J. P. , C. Oberlin , and E. Bey . 2007. “Acute Deep Hand Burns Covered by a Pocket Flap‐Graft: Long‐Term Outcome Based on Nine Cases.” Journal of Burns and Wounds 16, no. 6: e1. PMC1781957 17268577

[micr70104-bib-0034] Sarkar, A. , S. Raghavendra , M. G. Jeelani Naiyer , et al. 2014. “Free Thin Anterolateral Thigh Flap for Post‐Burn Neck Contractures—A Functional and Aesthetic Solution.” Annals of Burns and Fire Disasters 27, no. 4: 209–214. 26336369 PMC4544432

[micr70104-bib-0035] Schneider, J. C. , R. Holavanahalli , P. Helm , R. Goldstein , and K. Kowalske . 2006. “Contractures in Burn Injury: Defining the Problem.” Journal of Burn Care & Research: Official Publication of the American Burn Association 27, no. 4: 508–514. 10.1097/01.BCR.0000225994.75744.9D. 16819356

[micr70104-bib-0036] Sever, C. , F. Uygur , Y. Kulahci , H. Karagoz , and C. Sahin . 2012. “Thoracodorsal Artery Perforator Fasciocutaneous Flap: A Versatile Alternative for Coverage of Various Soft Tissue Defects.” Indian Journal of Plastic Surgery 45, no. 3: 478–484. 10.4103/0970-0358.105956. 23450715 PMC3580346

[micr70104-bib-0037] Slim, K. , E. Nini , D. Forestier , F. Kwiatkowski , Y. Panis , and J. Chipponi . 2003. “Methodological Index for Non‐Randomized Studies (Minors): Development and Validation of a New Instrument.” ANZ Journal of Surgery 73, no. 9: 712–716. 10.1046/j.1445-2197.2003.02748.x. 12956787

[micr70104-bib-0038] Sunil, N. P. , F. Ahmed , P. K. Jash , M. Gupta , and S. Suba . 2015. “Study on Surgical Management of Post Burn Hand Deformities.” Journal of Clinical and Diagnostic Research 9, no. 8: PC06–PC10. 10.7860/JCDR/2015/13316.6347. 26435994 PMC4576587

[micr70104-bib-0039] Uygur, F. , C. Sever , S. Tuncer , and Ş. Alagöz . 2009. “Reconstruction of Postburn Antebrachial Contractures Using Pedicled Thoracodorsal Artery Perforator Flaps.” Plastic and Reconstructive Surgery 123, no. 5: 1544–1552. 10.1097/PRS.0b013e3181a07439. 19407627

[micr70104-bib-0040] Verhaegen, P. D. H. M. , C. M. Stekelenburg , A. J. M. van Trier , F. B. Schade , and P. P. M. van Zuijlen . 2011. “Perforator‐Based Interposition Flaps for Sustainable Scar Contracture Release: A Versatile, Practical, and Safe Technique.” Plastic and Reconstructive Surgery 127, no. 4: 1524–1532. 10.1097/PRS.0b013e318208d1fb. 21460661

[micr70104-bib-0041] Vinh, V. Q. , R. Ogawa , T. Van Anh , and H. Hyakusoku . 2007. “Reconstruction of Neck Scar Contractures Using Supraclavicular Flaps: Retrospective Study of 30 Cases.” Plastic and Reconstructive Surgery 119, no. 1: 130–135. 10.1097/01.prs.0000244843.49596.e5. 17255666

[micr70104-bib-0042] Vinh, V. Q. , T. Van Anh , R. Ogawa , and H. Hyakusoku . 2009. “Anatomical and Clinical Studies of the Supraclavicular Flap: Analysis of 103 Flaps Used to Reconstruct Neck Scar Contractures.” Plastic and Reconstructive Surgery 123, no. 5: 1471–1480. 10.1097/PRS.0b013e3181a205ba. 19407618

[micr70104-bib-0043] Wang, X. K. , Q. K. Zhai , L. Xue , L. Lu , Y. X. Wang , and Z. L. Wang . 2012. “Treatment of Postburn Anteriorly Located Neck Contractures With Local Flaps.” Journal of Craniofacial Surgery 23, no. 5: e387–e390. 10.1097/SCS.0b013e31825882e7. 22976678

[micr70104-bib-0044] Woo, S. H. , and J. H. Seul . 2001. “Optimizing the Correction of Severe Postburn Hand Deformities by Using Aggressive Contracture Releases and Fasciocutaneous Free‐Tissue Transfers.” Plastic and Reconstructive Surgery 107, no. 1: 1–8. 11176593 10.1097/00006534-200101000-00001

[micr70104-bib-0045] Yildirim, S. , G. Avci , M. Akan , A. Misirlioğlu , and T. Aköz . 2003. “Anterolateral Thigh Flap in the Treatment of Postburn Flexion Contractures of the Knee.” Plastic and Reconstructive Surgery 111, no. 5: 1630–1631. 12655208 10.1097/01.PRS.0000055017.39339.C2

