# Supplementary material for: Outcomes of Microsurgical Reconstruction of Post‐Burn Joint Contracture—Systematic Review and Meta‐Analysis
Source: Microsurgery. 2025 Aug 15;45(6):e70104. doi: 10.1002/micr.70104 (PMC12357132; doi:10.1002/micr.70104)
Supplement: Supplementary file 1 — File S1: micr70104‐sup‐0001‐SDC1.docx. [file MICR-45-e70104-s001.docx]

| Burn contractures | Search: burn scar contracture (“burns” [MeSH Terms] OR “burns” [All Fields] OR “burn” [All Fields]) AND (“cicatrix” [MeSH Terms] OR “cicatrix” [All Fields] OR “scar” [All Fields]) AND (“contractural” [All Fields] OR “contracture” [MeSH Terms] OR “contracture” [All Fields] OR “contractures” [All Fields] OR “contractured” [All Fields]) Translations burn: “burns” [MeSH Terms] OR “burns” [All Fields] OR “burn” [All Fields] scar: “cicatrix” [MeSH Terms] OR “cicatrix” [All Fields] OR “scar” [All Fields] contracture: “contractural” [All Fields] OR “contracture” [MeSH Terms] OR “ contracture” [All Fields] OR “contractures” [All Fields] OR “contractured” [All Fields] |
| --- | --- |
