# Supplementary material for: Outcomes of Microsurgical Reconstruction of Post‐Burn Joint Contracture—Systematic Review and Meta‐Analysis
Source: Microsurgery. 2025 Aug 15;45(6):e70104. doi: 10.1002/micr.70104 (PMC12357132; doi:10.1002/micr.70104)
Supplement: Supplementary file 2 — File S2: micr70104‐sup‐0002‐SDC2.docx. [file MICR-45-e70104-s002.docx]

**List of Included Studies**

1. Davami, B., and G. Pourkhameneh. "Correction of Severe Postburn Claw Hand." Techniques in Hand & Upper Extremity Surgery, vol. 15, no. 4, 2011, pp. 260–264. <https://doi.org/10.1097/BTH.0b013e3182245b56>.
2. Chen, H. C., K. P. Wu, C. I. Yen, et al. "Anterolateral Thigh Flap for Reconstruction in Postburn Axillary Contractures." Annals of Plastic Surgery, vol. 79, no. 2, 2017, pp. 139–144. <https://doi.org/10.1097/SAP.0000000000001097>.
3. Angrigiani, C. "Aesthetic Microsurgical Reconstruction of Anterior Neck Burn Deformities." Plastic and Reconstructive Surgery, vol. 93, no. 3, 1994, pp. 507–518.
4. Wang, X. K., Zhai, Q. K., Xue, L., Lu, L., Wang, Y. X., & Wang, Z. L. (2012). Treatment of postburn anteriorly located neck contractures with local flaps. The Journal of craniofacial surgery, 23(5), e387–e390. <https://doi.org/10.1097/SCS.0b013e31825882e7>
5. Chang, L. S., Y. H. Kim, and S. W. Kim. "Reconstruction of Burn Scar Contracture Deformity of the Extremities Using Thin Thoracodorsal Artery Perforator Free Flaps." ANZ Journal of Surgery, vol. 91, no. 9, 2021, pp. E578–E583. <https://doi.org/10.1111/ans.16640>.
6. Karagoz, H., F. Eren, and E. Ulkur. "Use of the Lateral Intercostal Artery Perforator-Based Pedicled Reverse Thoraco-Abdominal Flap for Treatment of Antecubital Burn Contractures." Burns, vol. 37, no. 1, 2011, pp. 134–138. https://doi.org/10.1016/j.burns.2010.03.010.
7. Yildirim, S., G. Avci, M. Akan, A. Misirlioğlu, and T. Aköz. "Anterolateral Thigh Flap in the Treatment of Postburn Flexion Contractures of the Knee." Plastic and Reconstructive Surgery, vol. 111, no. 5, 2003, pp. 1630–1631.
8. El-Khatib, H. A., T. A. Mahboub, and T. A. Ali. "Use of an Adipofascial Flap Based on the Proximal Perforators of the Ulnar Artery to Correct Contracture of Elbow Burn Scars: An Anatomic and Clinical Approach." Plastic and Reconstructive Surgery, vol. 109, no. 1, 2002, pp. 130–136. https://doi.org/10.1097/00006534-200201000-00022.
9. Er, E., and C. Uçar. "Reconstruction of Axillary Contractures with Thoracodorsal Perforator Island Flap." Burns, vol. 31, no. 6, 2005, pp. 726–730. https://doi.org/10.1016/j.burns.2005.02.014.
10. Eski, M., M. Nisanci, and M. Sengezer. "Correction of Thumb Deformities after Burn: Versatility of First Dorsal Metacarpal Artery Flap." Burns, vol. 33, no. 1, 2007, pp. 65–71. https://doi.org/10.1016/j.burns.2006.04.030.
11. Feng, C. H., J. Y. Yang, S. S. Chuang, C. Y. Huang, Y. C. Hsiao, and C. Y. Lai. "Free Medial Thigh Perforator Flap for Reconstruction of the Dynamic and Static Complex Burn Scar Contracture." Burns, vol. 36, no. 4, 2010, pp. 565–571. https://doi.org/10.1016/j.burns.2009.07.005.
12. Gousheh, J., E. Arasteh, and P. Mafi. "Super-Thin Abdominal Skin Pedicle Flap for the Reconstruction of Hypertrophic and Contracted Dorsal Hand Burn Scars." Burns, vol. 34, no. 3, 2008, pp. 400–405. https://doi.org/10.1016/j.burns.2007.03.025.
13. Hafezi, F., B. Naghibzadeh, M. Pegahmehr, N. Boddouhi, and A. Nouhi. "Extended Vertical Trapezius Fasciocutaneous Flap (Back Flap) in Face and Neck Burn Scar Reconstruction." Annals of Plastic Surgery, vol. 61, no. 4, 2008, pp. 441–446. https://doi.org/10.1097/SAP.0b013e31815f128a.
14. Hassanpour, S. E., S. Motamed, and M. Ghazisaidi. "Treatment of Wide Scar Contracture of Antecubital Fossa with Bipedicle Flap from Scar Tissue." Burns, vol. 33, no. 2, 2007, pp. 236–240. https://doi.org/10.1016/j.burns.2006.06.023.
15. Nişanci, M., E. Er, S. Işik, and M. Sengezer. "Treatment Modalities for Post-Burn Axillary Contractures and the Versatility of the Scapular Flap." Burns, vol. 28, no. 2, 2002, pp. 177–180. https://doi.org/10.1016/S0305-4179(01)00090-0.
16. Sever, C., F. Uygur, Y. Kulahci, H. Karagoz, and C. Sahin. "Thoracodorsal Artery Perforator Fasciocutaneous Flap: A Versatile Alternative for Coverage of Various Soft Tissue Defects." Indian Journal of Plastic Surgery, vol. 45, no. 3, 2012, pp. 478–484. https://doi.org/10.4103/0970-0358.105956.
17. Uygur, F., C. Sever, S. Tuncer, and Ş. Alagöz. "Reconstruction of Postburn Antebrachial Contractures Using Pedicled Thoracodorsal Artery Perforator Flaps." Plastic and Reconstructive Surgery, vol. 123, no. 5, 2009, pp. 1544–1552. https://doi.org/10.1097/PRS.0b013e3181a07439.
18. Vinh, V. Q., R. Ogawa, T. Van Anh, and H. Hyakusoku. "Reconstruction of Neck Scar Contractures Using Supraclavicular Flaps: Retrospective Study of 30 Cases." Plastic and Reconstructive Surgery, vol. 119, no. 1, 2007, pp. 130–135. https://doi.org/10.1097/01.prs.0000244843.49596.e5.
19. Vinh, V. Q., T. Van Anh, R. Ogawa, and H. Hyakusoku. "Anatomical and Clinical Studies of the Supraclavicular Flap: Analysis of 103 Flaps Used to Reconstruct Neck Scar Contractures." Plastic and Reconstructive Surgery, vol. 123, no. 5, 2009, pp. 1471–1480. <https://doi.org/10.1097/PRS.0b013e3181a205ba>.
20. Loghmani, S., M. Eidy, M. Mohammadzadeh, A. Loghmani, and F. Raigan. "The Supraclavicular Flap for Reconstruction of Post-Burn Mentosternal Contractures." Iranian Red Crescent Medical Journal, vol. 15, no. 4, 2013, pp. 292–297. https://doi.org/10.5812/ircmj.1600.
21. Angrigiani, C., G. Artero, C. Sereday, et al. "Refining the Extended Circumflex Scapular Flap for Neck Burn Reconstruction: A 30-Year Experience." Journal of Plastic, Reconstructive & Aesthetic Surgery, vol. 70, no. 9, 2017, pp. 1252–1260.
22. Bali, Z. U., B. Özkan, Y. Keçeci, et al. "Reconstruction of Burn Contractures with Free Anterolateral Thigh Flap in Various Anatomic Sites." Ulusal Travma ve Acil Cerrahi Dergisi, vol. 27, no. 4, 2021, pp. 337–343.
23. Moroz, V., A. Yudenich, T. Kafarov, et al. "Reconstruction of Extensive Postburn Scar Deformities and Contractures of the Neck Using Expanded and Nonexpanded Free Tissue Transfer." European Journal of Plastic Surgery, vol. 24, 2001, pp. 217–220.
24. Mun, G. H., B. J. Jeon, S. Y. Lim, et al. "Reconstruction of Postburn Neck Contractures Using Free Thin Thoracodorsal Artery Perforator Flaps with Cervicoplasty." Plastic and Reconstructive Surgery, vol. 120, no. 5, 2007, pp. 1524–1532.
25. Sarkar, A., S. Raghavendra, M. G. Jeelani Naiyer, et al. "Free Thin Anterolateral Thigh Flap for Post-Burn Neck Contractures—A Functional and Aesthetic Solution." Annals of Burns and Fire Disasters, vol. 27, no. 4, 2014, pp. 209–214.
26. Woo, S. H., and J. H. Seul. "Optimizing the Correction of Severe Postburn Hand Deformities by Using Aggressive Contracture Releases and Fasciocutaneous Free-Tissue Transfers." Plastic and Reconstructive Surgery, vol. 107, no. 1, 2001, pp. 1–8.
27. Grishkevich V. M. (2012). Unilateral cervical burn scar deformity elimination with contralateral cervicothoracic flap--a new approach. Journal of burn care & research : official publication of the American Burn Association, 33(2), e26–e31. <https://doi.org/10.1097/BCR.0b013e3182331d4c>
